# The Centenary of Immune Thrombocytopenia—Part 2: Revising Diagnostic and Therapeutic Approach

**DOI:** 10.3389/fped.2017.00179

**Published:** 2017-08-21

**Authors:** Rita Consolini, Giorgio Costagliola, Davide Spatafora

**Affiliations:** ^1^Laboratory of Immunology, Department of Clinical and Experimental Medicine, Division of Pediatrics, University of Pisa, Pisa, Italy; ^2^Clinical Immunology and Allergy Unit, Department of Clinical and Experimental Medicine, University of Pisa, Pisa, Italy

**Keywords:** immune thrombocytopenia, differential diagnosis, diagnostic algorithm, chronic thrombocytopenia, conventional therapy, new therapeutic targets

## Abstract

Primary immune thrombocytopenia (ITP) is the most common cause of thrombocytopenia in children and adolescents and can be considered as a paradigmatic model of autoimmune disease. This second part of our review describes the clinical presentation of ITP, the diagnostic approach and overviews the current therapeutic strategies. Interestingly, it suggests an algorithm useful for differential diagnosis, a crucial process to exclude secondary forms of immune thrombocytopenia (IT) and non-immune thrombocytopenia (non-IT), which require a different therapeutic management. Advances in understanding the pathogenesis led to new therapeutic targets, as thrombopoietin receptor agonists, whose role in treatment of ITP will be discussed in this work.

## Introduction

The platelet threshold necessary to make diagnosis of thrombocytopenia has changed during years (1), and for immune thrombocytopenia (ITP) working group thrombocytopenia is defined as a condition characterized by a platelet count lower than 100,000 platelets per microliter (2).

Primary ITP is the most common cause of thrombocytopenia, having an estimated incidence of 4–9 cases out of 100,000 people per year (3–6), with about half of the pediatric cases occurring in previously healthy children.

The first part of this review described the pathogenesis of the disease, with special attention to the role of innate immune system and impairment in megakaryopoiesis (7). In this second part, we focus on the clinical aspects of ITP, particularly on the differential diagnosis and new target therapies. The process of differential diagnosis has the aim to distinguish ITP from secondary IT (caused by infections, immune defects, and other pathologies) and non-IT, particularly inherited diseases, because all these conditions require different treatments.

Therapy of ITP was historically based on the progressive use of immunoglobulins, corticosteroids, and immunosuppressive agents: following the recent advances in the study of the pathogenesis of the disease, new therapeutic targets have been identified, potentially leading to innovative therapeutic strategies.

## Classification

Immune thrombocytopenia can be classified according to etiology, disease evolution and age of onset. Etiologic classification will be discussed separately, to introduce the more common forms of secondary IT.

According to disease evolution, it is possible to identify three categories of ITP: newly diagnosed ITP; persistent ITP, still present after 3 months from diagnosis; and chronic ITP, lasting 12 or more months after diagnosis (2), which represents about 20% of the total cases of ITP in childhood (8).

IT affecting young children is typically acute and self-remitting, and primary forms are the most common in this age. Adolescents IT has an intermediate phenotype between childhood-onset and adult-onset forms (9), showing a higher rate of chronicity and a greater percentage of secondary IT.

## Etiology of ITP

Etiologic classification divides two categories of ITP: primary ITP and secondary IT.

The primary form of IT, classically defined “idiopathic,” is often seen in childhood and triggered by non-specific viral infections (upper respiratory or gastrointestinal infections): in some cases acute infections by Epstein–Barr virus, cytomegalovirus, parvovirus, rubella, mumps, and varicella have been identified as triggers of ITP (10–12).

Secondary IT has a complex etiology, as specific infections, drugs or vaccinations and immunologic abnormalities, including immunodeficiencies, can be involved in its pathogenesis.

### Infections

Infectious diseases caused by HIV, HCV, *Helicobacter pylori*, and dengue virus can be responsible of secondary IT, usually with chronic course (13–17), trough different mechanisms, like molecular mimicry, modulation of the immune system’s activity or suppression of bone marrow production (18). Association between pulmonary and extra-pulmonary TBC and IT is documented in only a few case reports (19, 20).

### Drugs

Secondary IT can be caused by the assumption of drugs and vaccines: drug-induced IT, ascribable in most of cases to the assumption of certain antibiotics, non-steroidal anti-inflammatory drugs, and antivirals, it is often not recognized, resulting in recurrent non-explained episodes of thrombocytopenia (21), that usually show a complete recovery after the withdrawal of the drug. Less commonly than in adulthood, it is possible to observe in childhood the development of heparin-induced thrombocytopenia (22).

Patients who received multiple transfusions are at risk for the development of posttransfusion purpura (PTP), a rare form of secondary IT with a high rate of bleeding: this is more frequent in multiparous female (18), but rare reports of PTP with pediatric onset are described (23).

### Immunodeficiency

Immune thrombocytopenia is a possible manifestation of immunodeficiency, particularly common variable immunodeficiency (CVID), selective IgA deficiency, and DiGeorge’s syndrome. Is interesting to underline that, in humoral immune defects, the reduction of platelet count may appear years before the hypogammaglobulinemia (24–26).

### Autoimmune Diseases

Systemic autoimmune diseases, such as systemic lupus erythematosus (SLE), Sjogren’s syndrome, and antiphospholipid syndrome, are associated with the development of IT (27): an isolated thrombocytopenia may represent the initial manifestation of SLE, preceding the diagnosis by several years (28, 29).

Current literature reports an association between IT and clinical and subclinical thyroid autoimmune diseases (Hashimoto’s and Basedow–Graves’ diseases), suggesting the presence of an overlap in the pathogenesis of these conditions (30, 31).

Moreover, many lymphoproliferative disorders may cause secondary ITP: in this category, the most frequent disease is autoimmune lymphoproliferative syndrome (32), mostly found in children aged under 3 years.

### Neoplasia

Lymphatic malignancies, particularly non-Hodgkin’s lymphoma, represent a cause of IT (33), which rarely can be a paraneoplastic manifestation of a solid neoplasia (34, 35), mostly in adults.

### Age-Related Considerations

In neonatal age, the most frequent form of IT is the alloimmune (36), caused by the production of maternal antibodies directed against platelet alloantigens.

In adolescent females, the possibility of an IT secondary to pregnancy should be considered (37).

## Other Causes of Thrombocytopenia in Pediatric Age

Other forms of thrombocytopenia occurring during childhood and adolescence could mime ITP and secondary IT, particularly when platelet reduction is the only laboratory finding. Inherited thrombocytopenias, often misdiagnosed as ITP (38), are characterized by impairment in magakaryopoiesis and include a large variety of X-linked and autosomal diseases, commonly presenting with altered platelet size (39). Among them, Wiskott–Aldrich syndrome, caused by the mutation of WAS gene on chromosome X, usually comprehends the association of thrombocytopenia with small platelets, eczema, and immunodeficiency (40).

Thrombocytopenia occurs also in acute leukemia and primary bone marrow failure syndromes as Fanconi anemia, but in these cases, the association with other cytopenias helps making differential diagnosis (41).

Also lisosomial storage disorders, as Gaucher’s and Niemann–Pick’s disease, may present thrombocytopenia at clinical onset, usually accompanied by a considerable splenomegaly (42, 43).

Other less common conditions are indicated in Table 1, which summarizes the most relevant causes of secondary IT and non-IT in childhood and adolescence.

**Table 1:** Etiology of secondary IT and non-IT in childhood and adolescence.

Secondary IT	Non-IT
Immunodeficiencies: CVID, IgA deficiency, DiGeorge’s syndrome Infections: HIV, HCV, CMV, EBV, *Helicobacter pylori*, and TBC Drugs: NSAIDS, antibiotics, and antivirals Vaccines: influenza, poliomyelitis, pneumococcal, MMR, HPV, and HBV (44) Posttransfusion purpura Connective tissue disease: LES, Sjogren, and APLS Autoimmune thyroiditis: Basedow’s and Hashimoto’s diseases Lymphoproliferative disorders: ALPS Neoplasia: LNH and solid tumors (paraneoplastic) Alloimmune (neonatal) Pregnancy-associated IT	Inherited disorders: autosomal dominant, autosomal recessive, and X-linked diseases Lisosomial storage disorders: Gaucher’s and Niemann–Pick’s disease Solid tumors with bone marrow infiltration (as neuroblastoma) (45) Hypersplenism and splenic sequestration Inherited and acquired bone marrow failure syndromes (MDS, AAA) (46) Acute leukemia Chemotherapy with bone marrow suppression (47) Disseminated intravascular coagulation (48) Thrombotic microangiopathy (HUS/TTP) (49) Kasabach–Merritt Syndrome (50)

*CVID, common variable immunodeficiency; MMR, measles, mumps, and rubella; ALPS, autoimmune lymphoproliferative syndrome; MDS, myelodisplastic syndrome; AAA, acquired aplastic anemia; HUS: hemolytic–uremic syndrome; TTP, thrombotic thrombocytopenic purpura; CMV, cytomegalovirus; EBV, Epstein–Barr virus; NSAIDS, non-steroidal anti-inflammatory drugs; IT, immune thrombocytopenia*.

## Clinical Presentation

Immune thrombocytopenia is not rarely asymptomatic, being observed during routinely laboratory evaluations. In symptomatic cases, the most common presenting feature is epistaxis (51), followed by cutaneous and mucosal minor bleeding.

Severe bleeding rates are more common in childhood compared to adult patients: a review by Neunert et al. showed that severe bleeding occurs in 20.2% of children and 9.6% of adults (52).

The most severe bleeding event is ITP-associated intracranial hemorrhage (ICH): it represents a rare cause of pediatric stroke (53), affecting only 0.4% of children with ITP (52), but complicated by elevated mortality rates, reported between 12 and 25% in recent studies (54–58). ICH, as other severe bleedings, occurs mostly in children with other features of bleeding and in patients with a platelet count lower than 10,000/μl (59) and is often preceded by a precipitant factor, like head trauma (55, 60, 61).

An anamnesis positive for previous minor bleeding is a risk factor for severe haemorrhagic events (52), while the clinical relevance of an occult hemorrhage, often identified in urinary tract, is not completely defined, as studies reported different conclusions about the association with future overt bleeding and ICH (55, 62).

## Diagnosis

Diagnosis of ITP remains one of exclusion, and differential diagnosis with secondary forms is crucial, because in these cases thrombocytopenia may be less responsive to conventional therapy, but only to the treatment of primary cause.

Interview should focus on the potential triggers of ITP (assumption of drugs, vaccines, and transfusions) and risk factors for secondary forms, as the presence of weight loss, chronic infections (HIV and HCV) and other immune-mediated disease. It is also important to investigate elements suggestive of inherited thrombocytopenia, as previous bleedings and positive familiar history.

During physical assessment, the clinician must search potential sites of bleeding (cutaneous and mucosal) and identify signs suggestive for secondary IT or other pathologies, by examining the presence of hepatosplenomegaly, abdominal masses, lymphoadenopathies, and bone pain.

Furthermore, we analyze the diagnostic the most relevant laboratory investigations in ITP, to introduce our diagnostic algorithm.

## Laboratory Investigations

### Full Blood Count with Citrate and Reticulocyte Determination

First laboratory step, has the role of excluding a pseudothrombocytopenia (EDTA related) (63) and the presence of other cytopenias. Patients with severe bleeding could show anemia, related to blood loss. In case of multiple cytopenias, diagnosis of ITP is unlikely and becomes mandatory to investigate for acute leukemia, lymphomas, bone marrow failure (aplastic anemia), and neoplastic infiltration of bone marrow.

### Mean Platelet Volume (MPV)

Mean platelet volume is useful in the first laboratory assessment and is normal or slightly high in patients with ITP, while it shows alterations (marco- or micro-thrombocythemia) in almost all patients with inherited thrombocytopenia. Due to the lack of validated thresholds, this exam is significant in presence of a great difference in MPV, as it happens in inherited thrombocytopenia, in which platelet volume can be 50–100% higher than normal values (64). In some particular cases, as mono- and biallelic Bernard–Soulier syndrome (65) and MYH-9-related disease, giant platelets may not be recognized by automatic counters, underestimating MPV and platelet count (66, 67).

### Peripheral Blood Smear

It may demonstrate alterations on red blood cells (for example, schistocytes in HUS/TTP) and white cells (blasts in leukemia), which exclude diagnosis of ITP (68). Moreover, analysis of blood smear could be useful to identify alterations of platelet size and correct measurement of their diameters. Many of congenital thrombocytopenias have also changes in platelet morphology, recognizable with blood smear (69). The interpretation of blood smear requires experience, and access may be limited in resource poor regions, making it not always applicable as a first-line tool.

### Reticulated Platelet Count (RPLT)

First described in 1969 (70), reticulated platelets are immature platelets circulating in the blood, containing a residual RNA. They can be analyzed with flow cytometry and give an indirect determination of thrombopoietic rate (71). Lack of standardization of methods and definition of threshold values make difficult the interpretation of RPLT value (72): even if the applicability of RPLT determination is not completely defined, recent works conclude that this is a promising tool to distinguish thrombocytopenia caused by bone marrow hypoproduction to that one due to platelet destruction (73). Moreover, a study by Thomas-Kaskel et al. demonstrated the correlation between reticulated platelet count and response to treatment (74).

### Rh (D) Typing

This exam should be performed in those patients candidate for receiving therapy with anti-D immunoglobulins (75–77). Currently, this treatment is no longer licensed in Europe (51), where Rh typing is not yet recommended.

### Autoantibodies

Antiplatelet antibodies showed absence of specificity for ITP, and therefore the determination is not routinely recommended (78). Other autoantibodies, particularly antinuclear (ANA) and antithyroid antibodies, may have a diagnostic role in identifying secondary IT or, respectively, patients at risk of developing chronic thrombocytopenia and thyroid diseases. Testing for these antibodies is particularly useful in patients with persistent or chronic ITP, as discussed in the Section “Prognosis and Sequelae.”

### Bone Marrow Examination

The analysis of bone marrow of a patient with ITP would show an increase in number of megakaryocytes and absence of alterations in other cellular lines. In patients with isolated thrombocytopenia, diagnosis of acute leukemia or lymphoma is unlikely, and bone marrow biopsy and aspirate are rarely useful. Several authors agree affirming that bone marrow biopsy and aspirate must be performed in children and adolescents with atypical findings for ITP (51, 79–81).

Moreover, bone marrow examination should be performed in patients with absence of response to standard treatments, before the beginning of second-line therapies and the execution of splenectomy (82).

## Algorithm for Differential Diagnosis

**Figure 1:**
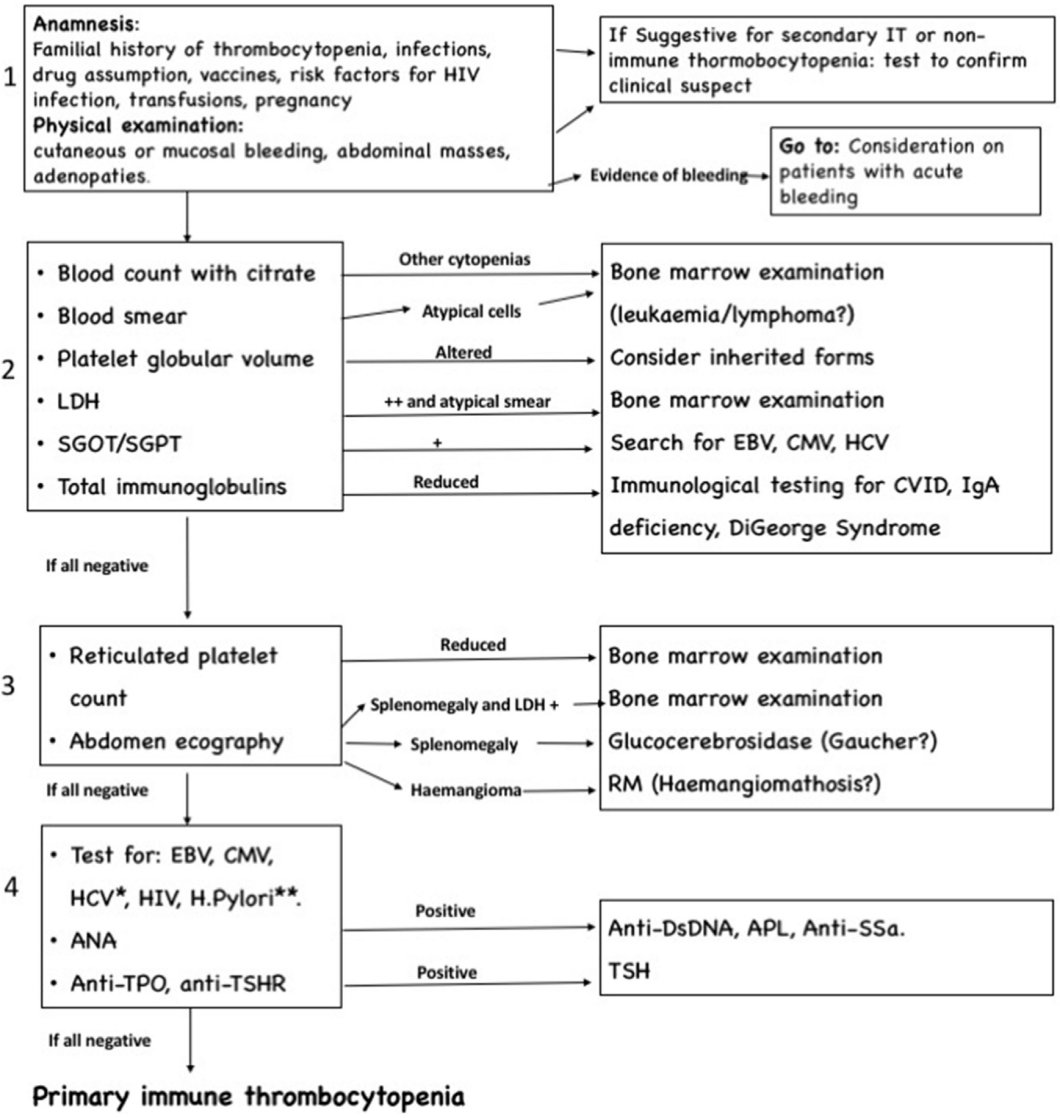
Algorithm for differential diagnosis of immune thrombocytopenia, composed by four progressive steps. *Testing for Epstein–Barr virus (EBV), cytomegalovirus (CMV), and HCV is recommended if not done in previous step. **Helycobacter pylori testing should be performed in high prevalence area or clinical suspect.

The algorithm we suggest for differential diagnosis of new onset thrombocytopenia, shown in Figure 1, is composed by one clinical step and three laboratory and radiological steps and is primarily directed to patients without acute and severe bleeding. We focused particularly on the exclusion of short-term life-threatening conditions, such as acute leukemia, lymphomas, and other neoplasia, while chronic infections and systemic autoimmune disease with partial expression are investigated in later steps. The algorithm is progressive, and therefore the investigations included in steps 3 and 4 are usually indicated only in patients with persistent and chronic ITP.

The clinical step remains fundamental: in case of severe bleeding signs, it is mandatory to treat the patient, and the required investigations are different (see Consideration on Patients with Acute Bleeding). Moreover, if anamnesis or physical assessment shows elements indicative for secondary IT (abdominal masses and adenopathies), the algorithm becomes not necessary, and the laboratory and radiological approach must start with investigations directed to confirm the etiologic hypothesis suggested by the clinical findings.

The second step comprehends laboratory exams directed to identify the conditions that more frequently cause secondary IT and non-ITP, including inherited thrombocytopenia, infections, immunodeficiency, and lymphoid malignancies.

The third step includes an abdominal echography, useful to recognize alterations in liver, spleen, and abdominal lymph nodes, not always appreciable during clinical examination. This step also considers the determination of reticulated platelet count: despite the lack of standardization of values and difficulties in interpretations, this investigation, when available, can give important information about thrombopoietic rate, and thus remains an option to consider.

The last step comprehends investigations for autoimmune diseases and chronic infections. Determination of ANA is also important to predict the evolution to a chronic form (see Prognosis and Sequelae).

### Consideration on Patients with Acute Bleeding

In this case, primary diagnostic approach should exclude conditions, such as HUS/TTP, DIC, antiphospholipid syndrome, coagulation abnormalities, and neoplasia (promyelocytic leukemia). First-step analyses include determination of full blood count, blood smear (if available), coagulation tests, APL, LDH, and D-dimer, accompanied by the evaluation of the bleeding site (echography, endoscopy, and neuroimaging). In case of negative results or resolution of the bleeding episode, it is possible to apply the diagnostic algorithm discussed above.

## Therapy

About two out of three pediatric patients with ITP show a spontaneous improvement in platelet count in 6 months without necessity of medical treatment, and those remissions are usually sustained. Most of patients with newly diagnosed ITP do not show signs of bleeding, and can be managed with a “watch and see” strategy (83–86).

There is no absolute consensus about the platelet threshold necessary to start treatment in ITP: 1996 guidelines of the American Society of Hematology recommended to treat patients with a platelet count lower than 10,000/μl and minor purpura or those one with a count lower 20,000/μl and significant bleeding (87). An update published in 2011 suggested that children without bleeding or with mild bleeding should be managed only with observations, regardless of platelet count (88). Despite these recommendations, most patients with low risk of bleeding are currently treated (89).

### First-line Treatment

#### Prednisone–Prednisolone

All guidelines support the use of corticosteroids in the first-line treatment of ITP. Oral prednisone is often effective in inducing response in pediatric patients when administered at doses of 1–2 mg/kg for 7–14 days and maintains efficacy also at higher doses (4 mg/kg/day) for 3 or 4 days, raising platelet count over 50,000/μl in the first 72 h in 72–88% of patients (78, 90, 91).

However, due to the adverse effects of a prolonged treatment with corticosteroids in children, those drugs must be used only for short periods, to maintain a hemostatic platelet count (78).

#### Intravenous Immunoglobulins (IVIg)

Immunoglobulins have been used for ITP since 1981 (92, 93), for the effect of modulation on immune system. The treatment induces a raise in platelet count in 80% of pediatric patients, obtaining an effect in the first 48 h more frequent than corticosteroids (94). IVIg are usually administered in a single dose of 0.8–1 g/kg, with the chance of using a second dose in case of incomplete response, even if also lower doses (0.6 g/kg) are reported to be effective (95). Adverse effects include headache and fever and are more common when used doses are greater than 1 g/kg for consecutive days (91).

#### Intravenous Anti-D Immunoglobulin

Rh-positive children could receive short infusions of anti-D immunoglobulin, with a recommended dose of 50–75 µg/kg (78). This therapeutic strategy has a response rate greater than 50% and acts more rapidly than IVIG (76, 77, 96, 97).

However, in patients with comorbidity, the treatment has been associated with severe hemolysis, acute renal failure, and disseminated intravascular coagulation, and therefore anti-D immunoglobulin administration should require a careful selection of patients and post-therapy monitoring, as concluded by Despotovic et al. (98).

### Second-line Therapies

#### High-Dose Corticosteroids

High-dose methylprednisolone has been used as an alternative to IVIg, showing comparable response rates (99, 100).

Dexamethasone (28–40 mg/m^2^/day) has been used in pediatric patients with chronic refractory ITP, obtaining response rates greater than 80%, with and a mean duration of the response of 26 months (101): moreover, psychiatric adverse effects, such as insomnia and aggressive behavior, are extremely frequent (102), and this makes dexamethasone only a second-line therapeutic alternative.

#### Rituximab

This anti-CD20 antibody, used in other autoimmune diseases and B-cell lymphoma, has been used in chronic refractory ITP often showing response rates greater than 60% (103–106), even though in a study by Bennett et al. only 31% of patients responded (107). However, follow-up studies have shown that sustained response is uncommon (108, 109), and safety profile is unclear.

#### Danazol

This attenuated androgen is successfully used in second-line treatment of adult patients with ITP, particularly in elderly patients (110). There are only a few studies about its use in pediatric patients, showing a good effectiveness without significant adverse reactions. Unfortunately, danazol can accelerate bone growth, and this limits its applicability in prepuberal patients (111, 112).

#### Thrombopoietin Receptor Agonists (TPO-RAs)

Since the discovery of the role of thrombopoietin (TPO) in ITP several thrombopoietic drugs was tested (113), and in 2008 FDA approved two TPO receptor agonists for non-responsive ITP in adults: romiplostim and eltrombopag (114, 115). Romiplostim acts on TPO-binding subunit of the receptor and is administered subcutaneously weekly (116). It is not yet approved for childhood-onset ITP, although in several studies it showed a 50–80% response rate, without severe adverse effects (117–125).

Eltrombopag acts binding the transmembrane domain of TPO receptor and is administered orally daily (116). It showed response rates greater than 60% in two randomized trials, associated with a good tolerability (126, 127), so in 2015 FDA has approved it for the use in childhood-onset disease. Reported adverse effects consist in an increased risk of hepatic damage and cataract.

Recently, avatrombopag, a new drug with an eltrombopag-like mechanism of action, was included in clinical trials for adults, showing response rate similar to other TPO-RAs in absence of severe adverse effects (128). In summary, TPO-RAs seem to be safe end effective also in childhood-onset refractory ITP.

#### Use of Immunosuppressors

There are only a few studies investigating the role of immunosuppressive agents, single or in combination, in patients with refractory ITP, and experience in childhood is not enough strong to give specific recommendations (78). Azathioprine, used in several autoimmune pediatric diseases, is still an option for the treatment of adult patients with ITP, particularly in chronic ITP and when splenectomy is contraindicated or has been ineffective (129). Response is detectable after about 4 months, and adverse effects, such as posttreatment leukemia, are extremely rare (130).

In pediatric age, cyclosporine is used in several conditions (organ transplants, autoimmune hepatitis, acquired aplastic anemia, juvenile dermatomyositis, and nephrotic syndrome) while its applicability in ITP is not completely defined. In adult patients, this drug often shows positive response rates both in single therapy and in association with steroids, with possibility of sustained remission after discontinuation of treatment (131, 132). Despite the lack of evidence in childhood-onset ITP and the necessity of further studies, these data support the potential utility of immunosuppressive agents as a second-line treatment in refractory ITP.

#### Splenectomy

Several studies reported a response in almost 85% of patients after splenectomy, with a 20–25% of relapses during the following years (133–135). Many works investigated the role of potential predictors of response to splenectomy in children and adults and suggested that female sex, younger age, response to steroids, and higher platelet count could be positive prognostic determinants, although the role of response to steroids is not univocally accepted (135–141).

Patients who received splenectomy are at risk of developing relevant adverse effects, particularly infections and sepsis by capsulated bacteria, with reported mortality rates of 3% (133), and thus the procedure is rarely recommended in children (78), being usually performed only in selected cases.

### New Therapeutic Targets

**Figure 2:**
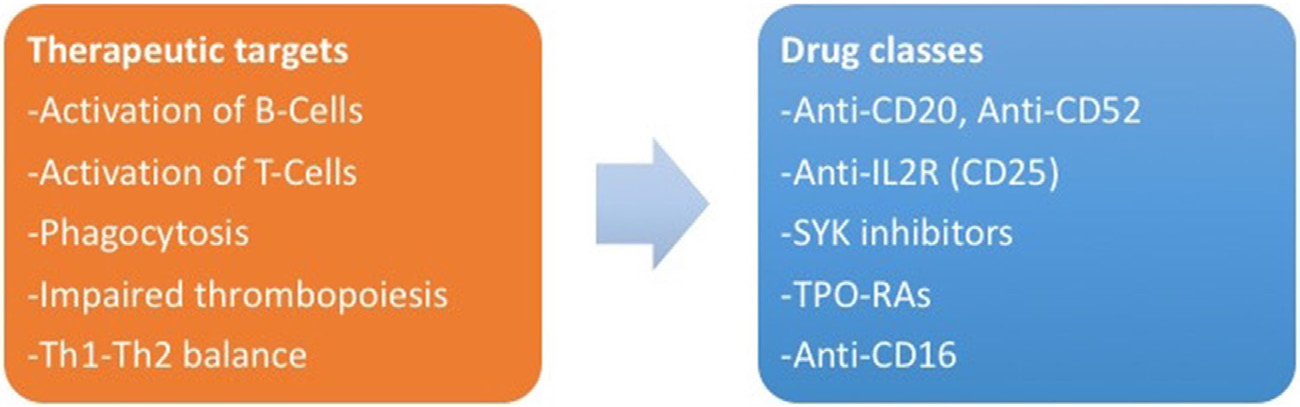
(Left) The new therapeutic targets identified by the study of pathogenesis of immune thrombocytopenia. (Right) Corresponding drug classes.

There are ongoing trials about other classes of drugs for ITP, currently limited to application in adulthood. New potential targets are represented by interaction between T-cells and antigen-presenting cells (anti-CD40L antibodies) (142), platelet phagocytosis [SYK inhibitors and interference with FcR binding on macrophages (143)], activation of B-cells (anti-CD52 or alemtuzumab) (144) and T-cells [anti-IL-2R or daclizumab (145)], and TH1 expansion (anti-CD16) (146, 147). Figure 2 summarizes the new therapeutic targets for ITP and the corresponding drug classes.

### Emergency Treatment in Childhood

In both adult and childhood-onset disease, in cases of life-threatening hemorrhage or organ damage, it is necessary to obtain a rapid raise in platelet count, to reduce bleeding risk. Consensus document of 2010 recommends to administer a high dose of platelet (two to three times larger than usual), accompanied with intravenous corticosteroids and IVIg. In particular cases, such as unstoppable bleeding, it is possible to consider the execution of a splenectomy in emergency (78).

## Prognosis and Sequelae

Immune thrombocytopenia in childhood is usually self-remitting, while there is the possibility of reactivation of the disease following viral infections or other triggers. The major sequelae of acute episodes are represented by permanent neurologic damages, defined as epilepsy, cognitive and learning disorder, and paresis, that can be detected in patients surviving the acute event of ICH (55, 148).

Adolescents are more likely to develop a chronic disease: a recent work by Heitink-Pollè et al. identified some predictors of chronic ITP, including female sex, age >11, insidious onset, absence of a trigger (infection or vaccination), and ANA positivity (149).

Patients with chronic ITP can undercover secondary misdiagnosed forms, as chronic infections, CVID, or other autoimmune pathologies, suggesting that this subset of patients need continuous reevaluation and discussion of primary diagnosis. Moreover, rarely, these patients may show an evolution to SLE: since there are no specific predictors of this progression (150), a study by Panzer et al. suggested that combined assessment of ANA and anti-DsDNA may have a role in identifying subjects at higher risk (151), who need to be periodically monitored.

## Conclusion

The centenary of ITP traced history characterized of new progressive knowledge, making it a paradigmatic model of autoimmune disease. In this issue, a second part of a revision of ITP story, we focused on ITP diagnostic approach. By combining physical examinations and laboratory findings, we designed a diagnostic algorithm, to dissect the complex diagnosis, substantially based on the exclusion of the multiple possible concurrent causes of thrombocytopenia. We described conventional therapy of ITP and focused on the new therapeutic targets.

## Author Contributions

All the authors contributed to the work presented in this paper, wrote and reviewed the paper, and provided approval of the final version.

## Conflict of Interest Statement

The authors declare that the research was conducted in the absence of any commercial or financial relationships that could be construed as a potential conflict of interest.
